# Avian Paramyxovirus Type-3 as a Vaccine Vector: Identification of a Genome Location for High Level Expression of a Foreign Gene

**DOI:** 10.3389/fmicb.2017.00693

**Published:** 2017-04-20

**Authors:** Asuka Yoshida, Siba K. Samal

**Affiliations:** Virginia-Maryland Regional College of Veterinary Medicine, University of Maryland, College ParkMD, USA

**Keywords:** RNA viruses, avian paramyxovirus serotype-3, foreign gene expression, intergenic region, optimal insertion site

## Abstract

Avian paramyxovirus serotype 3 (APMV-3) causes infection in a wide variety of avian species, but it does not cause apparent diseases in chickens. On the contrary, APMV-1, also known as Newcastle disease virus (NDV), can cause severe disease in chickens. Currently, natural low virulence strains of NDV are used as live-attenuated vaccines throughout the world. NDV is also being evaluated as a vaccine vector against poultry pathogens. However, due to routine vaccination programs, chickens often possess pre-existing antibodies against NDV, which may cause the chickens to be less sensitive to recombinant NDV vaccines expressing antigens of other avian pathogens. Therefore, it may be possible for an APMV-3 vector vaccine to circumvent this issue. In this study, we determined the optimal insertion site in the genome of APMV-3 for high level expression of a foreign gene. We generated recombinant APMV-3 viruses expressing the green fluorescent protein (GFP) by inserting the GFP gene at five different intergenic regions in the genome. The levels of GFP transcription and translation were evaluated. Interestingly, the levels of GFP transcription and translation did not follow the 3′-to-5′ attenuation mechanism of non-segmented, negative-sense RNA viruses. The insertion of GFP gene into the P-M gene junction resulted in higher level of expression of GFP than when the gene was inserted into the upstream N-P gene junction. Unlike NDV, insertion of GFP did not attenuate the growth efficiency of AMPV-3. Thus, APMV-3 could be a more useful vaccine vector for avian pathogens than NDV.

## Introduction

Among the members of the order *Mononegavirales*, the family *Paramyxoviridae* includes pathogenic and non-pathogenic viruses whose natural hosts include avian and aquatic animals as well as humans (Falk et al., 2008; Nylund et al., 2008; Lamb and Parks, 2013). Members of this family are primarily characterized by possessing a linear, non-segmented negative-sense (NNS) RNA genome, which encodes 6–10 viral genes tandemly (Lamb and Parks, 2013). Some paramyxoviruses, such as Sendai virus, human parainfluenza virus, and Newcastle disease virus (NDV), have been used as viral vectors in biological experiments, vaccine development and cancer therapy, and offer several advantages (Yonemitsu and Kaneda, 1999; Matveeva et al., 2015; Kim and Samal, 2016; Liang et al., 2016; Seki and Matano, 2016; Swett-Tapia et al., 2016; Yamasaki et al., 2016). First, the complete cytoplasmic replication of paramyxoviruses limits their integration into host cell genome. Secondly, the genome of paramyxoviruses does not undergo recombination, which makes these vectors genetically stable. Furthermore, paramyxoviruses can replicate in different cell lines and also can infect several animal species, which allows their use as viral vectors in different animal species. Additionally, replication of paramyxoviruses is generally limited to the respiratory tract, and do not spread to other organs. Hence, these viruses are highly safe to use as viral vectors (Lamb and Parks, 2013).

The avian paramyxoviruses are classified in the genus *Avulavirus* and are divided into 13 serotypes based on hemagglutination inhibition (HI) and neuraminidase inhibition (NI) assays (Afonso et al., 2016; Goraichuk et al., 2016). All strains of NDV belong to avian paramyxovirus serotype-1 (APMV-1) (Alexander, 1980). NDV causes a broad range of clinical disease in chickens resulting in an enormous economic loss to the poultry industry. NDV strains are categorized into three pathotypes based on the severity of the disease in chickens: lentogenic (low virulent), mesogenic (moderately virulent), and velogenic (highly virulent) (Alexander, 2000). Lentogenic NDV strains have been widely used as live vaccines for the poultry. By taking advantage of the reverse-genetic technique, NDV-based recombinant vaccines have been generated and reported to be able to induce protective immune responses against avian and non-avian disease-causing viruses (DiNapoli et al., 2009, 2010; Khattar et al., 2010, 2015; Kortekaas et al., 2010; Xiao et al., 2011; Kong et al., 2012; Kanabagatte Basavarajappa et al., 2014; Kim et al., 2014a,b; Kim and Samal, 2016; Yu et al., 2016). However, commercial chickens often possess pre-existing antibodies including maternal antibodies against NDV due to routine vaccination programs (Poissonnier and Teissier, 2013), resulting in a potential less-sensitivity to recombinant NDV vaccines expressing antigens of other avian pathogens.

The development of other APMV serotypes as vaccine vectors would circumvent the issue of pre-existing antibodies to NDV in commercial chickens. Among other APMV serotypes, APMV-3 is an attractive vaccine vector because it replicates well in cell lines and also in chickens and turkeys (Kumar et al., 2010; Kim et al., 2012). Since the 1960’s, many APMV-3 strains have been isolated from turkey (Alexander and Chettle, 1978; Tumova et al., 1979; Alexander et al., 1983; Macpherson et al., 1983). One of the strains, strain Netherland (GenBank: EU403085.1), has a genome size of 16,722 nucleotides (nt) long, containing six genes tandemly in a non-overlapping form (Kumar et al., 2008). The F protein cleavage site of the APMV-3 strain Netherland (ARPRGR↓L) lacks the dibasic furin motif (RX[R/K]R↓) required for intracellular cleavage, thus, the virus requires supplementation of exogenous protease to replicate *in vitro* (Kim et al., 2012). Unlike APMV-1, the pathogenesis of this virus has not been well characterized in domestic poultry. Although APMV-3 causes negligible disease associated with loss of egg production in turkeys and chickens (Kumar et al., 2010; Kim et al., 2012), it has been shown to be less virulent than the highly attenuated vaccine strains of NDV with an intracerebral pathogenicity index (ICPI) of 0.0 (Kumar et al., 2011). We previously reported that despite the poor replication of recombinant APMV-3 viruses expressing either NDV F or HN protein in chickens, infection resulted in the induction of neutralizing antibodies against NDV and provided protection against NDV challenge (Kumar et al., 2011).

Non-segmented negative-sense RNA viruses have been shown to express a foreign protein when the foreign gene is inserted into the non-coding region at a gene-junction in the viral genome (Daito et al., 2011; Samal, 2011; Luo et al., 2016). Due to single polymerase entry site of NNS RNA viruses, the promoter-proximal gene is transcribed in greatest amount, and each successive downstream gene is transcribed in progressively lower amount (Lamb and Parks, 2013). Previous studies have shown different expression levels of foreign genes depending on the insertion position in the genome of NNS RNA virus (Daito et al., 2011; Tomczyk and Orzechowska, 2013; Kim and Samal, 2016; Luo et al., 2016). In some cases, the addition of a foreign gene has little or no effect on replication of the virus, but in other cases the insertion of the foreign gene can cause a dramatic decrease on replication of the virus (Zhao et al., 2015). In this study, we have inserted the green fluorescent protein (GFP) gene at each of the five different gene junctions of the APMV-3 genome and compared the effect of the foreign gene insertion on viral replication and level of the expression. Our results showed that the level of GFP expression did not follow the 3′-to-5′ attenuation, since the insertion of GFP gene into the P-M gene junction resulted in an expression level higher than that when the gene was inserted into the upstream N-P gene junction. However, insertion at all positions in the genome stably maintained expression of the GFP gene. Analysis of the replication of the viruses showed that the virus containing N-P insertion had a slightly delayed growth kinetics compared to other viruses. These results further our understanding of transcriptional control of NNS viruses. The results presented here have direct relevance to the use of APMV-3 as a vaccine vector. We have identified the optimal insertion site in the genome of APMV-3 for foreign gene expression and also shown that all positions of insertion are stable for expression of a foreign gene.

## Materials and Methods

### Cells, Viruses, and Antibodies

HEp-2 cells (human epidermoid carcinoma) and DF-1 cells (chicken embryo fibroblast) were obtained from the American Type Culture Collection (Manassas, VA, USA). The cell lines were maintained in Dulbecco’s minimal essential medium (DMEM) supplemented with 10% FBS. A modified vaccinia virus Ankara strain (MVA) expressing T7 RNA polymerase was kindly provided by Dr. Bernard Moss (NIH, Bethesda, MD, USA) and propagated in primary chicken embryo fibroblast cells in DMEM with 2% FBS. Polyclonal antibody (pAb) against GFP, horseradish peroxidase (HRP)-conjugated anti-rabbit IgG goat antibodies (Abs) (Rockland Immunochemical Inc. and KPL, respectively), Alexa Fluor 546-conjugated anti-rabbit IgG Abs (Thermo Fisher Scientific) were used according to the protocols of the suppliers.

### Construction and Recovery of Recombinant Viruses

**FIGURE 1:**
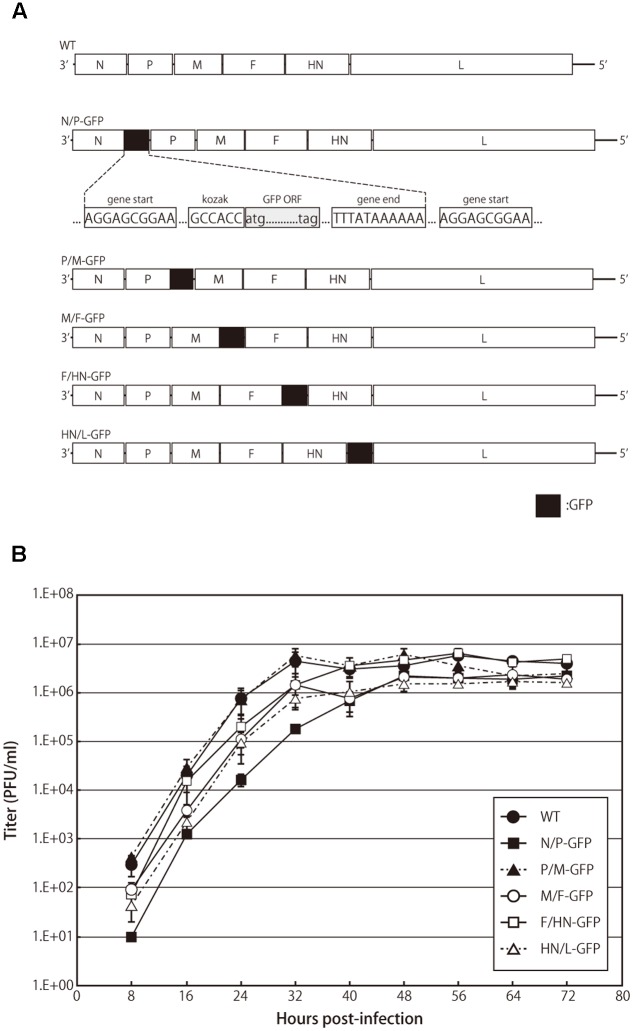
**Recombinant avian paramyxovirus serotype 3 (APMV-3) viruses and their growth kinetics. (A)** Schematic representation of parental virus and recombinant APMV-3 viruses expressing green fluorescent protein (GFP). GFP gene was flanked by gene start and gene end signals of APMV-3 and inserted into indicated intergenic regions of an APMV-3 antigenomic cDNA. The genes derived from APMV-3 and GFP are shown as white and black, respectively. **(B)** Multicycle growth kinetics of parental and recombinant APMV-3 viruses. DF-1 cells cultured in 6-well plates were infected with parental and recombinant viruses at an MOI of 0.01. Supernatants were collected at 8 h intervals until 72 h p.i. and virus titers were determined at each time point by plaque assay. Each titer represents the average of three independent experiments.

The GFP cDNA flanked with the APMV-3 gene start and gene stop sequence motifs were amplified using specific primers and inserted into the indicated gene junctions in the full-length cDNA genome clone of APMV-3 (**Figure 1**). All recombinant viruses were recovered as described previously (Huang et al., 2001). The presence of inserted GFP gene was confirmed by DNA sequencing of the RT-PCR products using viral genomic RNAs isolated from purified virions. Viral titers were determined and represented as the number of plaque-forming units (PFU)/ml, as described previously (Huang et al., 2001).

### Multi-step Growth Curves of APMV-3 Recombinants

DF-1 cells cultured in 6-well plates were infected with APMV-3 recombinant viruses at a multiplicity of infection (MOI) of 0.01. After 1-h incubation at 37°C, inocula were removed, cells were washed with PBS three times, and were incubated with DMEM containing 2% FBS and 10% allantoic fluid at 37°C for 72 h. Culture medium was harvested at the indicated time points, and titrated as described above.

### RNA Preparation

DF-1 cells cultured in 6-well plates were infected with APMV-3 recombinant viruses at an MOI of 0.5. At 24 h post-infection (p.i.), cells were harvested, and total RNA was isolated using an RNeasy Mini Kit (QIAGEN). mRNAs were isolated from the total RNA using Oligotex mRNA Mini Kit (QIAGEN).

### Analysis of GFP Expression

DF-1 cells cultured in 6-well plates were infected with APMV-3 recombinant viruses at an MOI of 0.5. At 24 h p.i., cells were immunostained with anti-APMV-3 pAb as primary Abs, and Alexa Fluor 546-conjugated anti-rabbit IgG Abs as secondary Abs. GFP fluorescence was visualized under a fluorescent microscope. DF-1 cells cultured in 6-well plates were infected with APMV-3 recombinant viruses at an MOI of 0.5. At 12, 24, and 48 h p.i., cells were harvested and suspended in SDS–PAGE sample buffer (125 mM Tris-HCl [pH 6.8], 4.6% SDS, 10% 2-mercaptoethanol, 0.005% bromophenol blue and 20% glycerol). The amount of GFP in infected cells at different time points was also analyzed by SDS–PAGE and followed by Western blotting with anti-GFP pAb.

### RT-PCR and Quantitative RT-PCR

All the primers used to detect anti-genomic RNA or mRNA and their sequences are provided in **Table 1**. Primers APMV-3 412F, APMV-3 1916F, APMV-3 8423F, APMV-3 721R, APMV-3 2225R, and APMV-3 8732R are complementary to the regions 412–436, 1916–1939, and 8423–8446 of the (-)-sense genome, and 698–721, 2202–2225, and 8708–8732 of the (+)-sense genome of APMV-3, respectively. Primers GFP 235F and GFP 515R are complementary to the region 235–258 of (-)-sense RNA, and 492–515 (+)-sense RNA, respectively. For the two-step RT-PCR, first-strand cDNAs were synthesized by SuperScript IV Reverse Transcriptase (Thermo Fisher Scientific) using the RNA samples prepared as described before and the APMV-3 721R, APMV-3 2225R, APMV-3 8732R, and GFP 515R primers were used to detect (+)-sense RNAs, respectively. The cDNAs were applied to quantitative real-time PCR (qPCR) using a SYBR Select Master Mix for CFX (applied biosystems) with a primer set of APMV-3 412F + APMV-3 721R, APMV-3 1916F + APMV-3 2225R, APMV-3 8423F + APMV-3 8732R, and GFP 235F + GFP 515R and analyzed using a CFX Connect^TM^ Real-Time PCR Detection System (Bio-Rad). Amplification of a specific cDNA fragment during the cycle reaction was confirmed by measuring the melting temperature of the PCR product.

**Table 1:** Oligonucleotide primers used in qRT-PCR.

Primer	Product length (bp)	Nucleotide sequence (5′ > 3′)	GC (%)
APMV-3 412F	310	AGTTGTGGACTTTGATGGACTTGAG	44.0
APMV-3 721R		TCGCCTGTCAATTCTATTCTGTTG	41.6
APMV-3 1916F	310	AATCACAAAGCCAGTGTCGATAAG	41.6
APMV-3 2225R		ATTCCGAAAAGTTGAGCGCTTTGC	45.8
APMV-3 8423F	310	TATGGTTACTCCGTAACGGAGATG	45.8
APMV-3 8732R		CACCACTTAAATTCCCCTATTGTAC	40.0
GFP 235F	281	TATGGTTACTCCGTAACGGAGATG	45.8
GFP 515R		GTACAATAGGGGAATTTAAGTGGTG	45.8

*GC, guanine-cytosine*.

## Results

### Growth Characteristics of APMV-3 Recombinants

We have previously reported the expression of foreign genes introduced between the P and M genes of recombinant APMV-3 genome (Kumar et al., 2011). In this study, we have compared the expression efficiency of the same foreign gene inserted at different gene junctions of the recombinant APMV-3 genome. The GFP gene flanked by the transcription start and stop signal sequences of APMV-3 was inserted into the indicated gene junctions of APMV-3 genomic cDNA (**Figure 1A**). All recombinant viruses were successfully recovered and passaged four times in embryonated chicken eggs. The multicycle growth kinetics of all recombinant viruses was compared in DF-1 cells. Each titer represents the average of three independent experiments (**Figure 1B**). At the final time point, there were no significant difference in the viral titers between the recombinant viruses expressing GFP and the parental virus, which reached 1.6–4.9 × 10^6^ PFU/ml (**Figure 1B**). However, at early time points, the recombinant virus with the GFP gene inserted between the N and P genes, had slower growth kinetics compared to the other recombinant viruses and the parent virus (**Figure 1B**). These results indicated that the GFP insertion do not largely affect virus growth except when it is inserted at the N and P gene junction.

### Insertion of GFP Gene into the P-M Gene-junction Maximizes the Level of Expression

**FIGURE 2:**
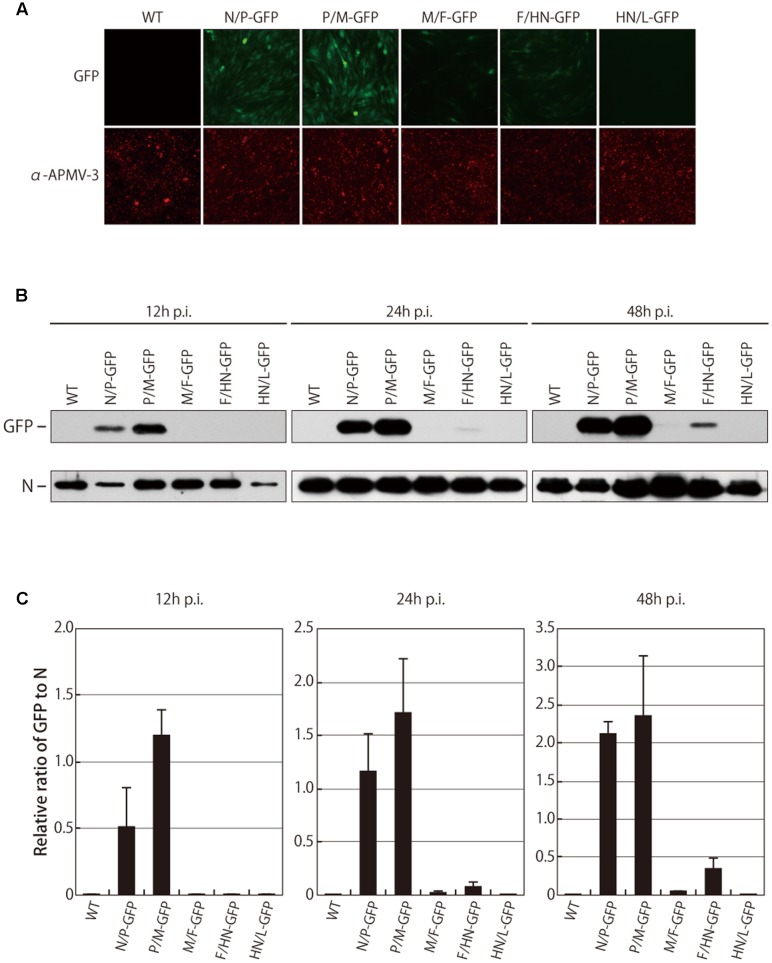
**Fluorescent intensity and cellular expression of GFP. (A)** DF-1 cells were infected with indicated viruses. After 24 h, cells were immunostained with the anti-N APMV-3 pAb, and the intensity of GFP fluorescent was analyzed under a microscope. **(B)** The level of GFP expression in cells infected with indicated viruses was analyzed by Western blot at indicated time points. **(C)** Amount of N proteins and GFP proteins were quantitated and the ratio of GFP to N is shown as bar graph.

To evaluate the levels of GFP expression, DF-1 cells were infected with the recombinant viruses at an MOI of 0.5, and the intensity of the GFP fluorescence was measured by fluorescence microscopy at 24 h p.i.. Unexpectedly, the maximum fluorescence of GFP was observed when the gene was placed between the P and M genes, but not between the upstream N and P genes (**Figure 2A**). Except for the P/M-GFP virus, the GFP expression was gradually reduced according to the GFP position in the genome in a 3′-to-5′ attenuation manner. The GFP expression of the P/M-GFP virus was 2.3-fold higher than the N/P-GFP virus (data not shown), although the GFP was inserted more upstream in the N/P-GFP virus than in the P/M-GFP virus.

The relative amount of GFP expressed in the infected cells was also analyzed by Western blot. The levels of viral N protein were identical among all the recombinant viruses, confirming that the replication of all of the viruses was similar. As expected, the GFP expression levels in the cells infected with the M/F-GFP and F/HN-GFP virus were reduced by 53.9-fold and 15.0-fold at 24 h p.i. and 45.4-fold and 6.3-fold at 48 h p.i. compared to the cells infected with the N/P-GFP virus, respectively (**Figures 2B,C**). However, the GFP expression level in the cells infected with the P/M-GFP virus was higher than the expression level of all the other viruses, including the N/P-GFP virus, at several time points (**Figures 2B,C**). The possibility that the increase in GFP expression exhibited by the P/M-GFP virus was caused by unwanted mutation in the genome of the virus was excluded since no mutation was found throughout the entire genome of the P/M-GFP virus. In order to further confirm that there was no mutation in the genome of the remaining four recombinant viruses, the beginning of the N gene to the beginning of the L gene of these viruses was sequenced. Our results did not find any mutations in the genome of these recombinant viruses, indicating that the level of GFP expression of these viruses could not have been affected by any unwanted mutation in the genome. Taken together these observation, strongly suggest that the P-M gene junction is the optimal insertion site in APMV-3 vector to obtain the highest level of expression of a foreign gene.

### Transcription of a Foreign GFP Gene Is Maximized in the P-M Gene Junction Rather than in the Upstream N-P Gene Junction

**FIGURE 3:**
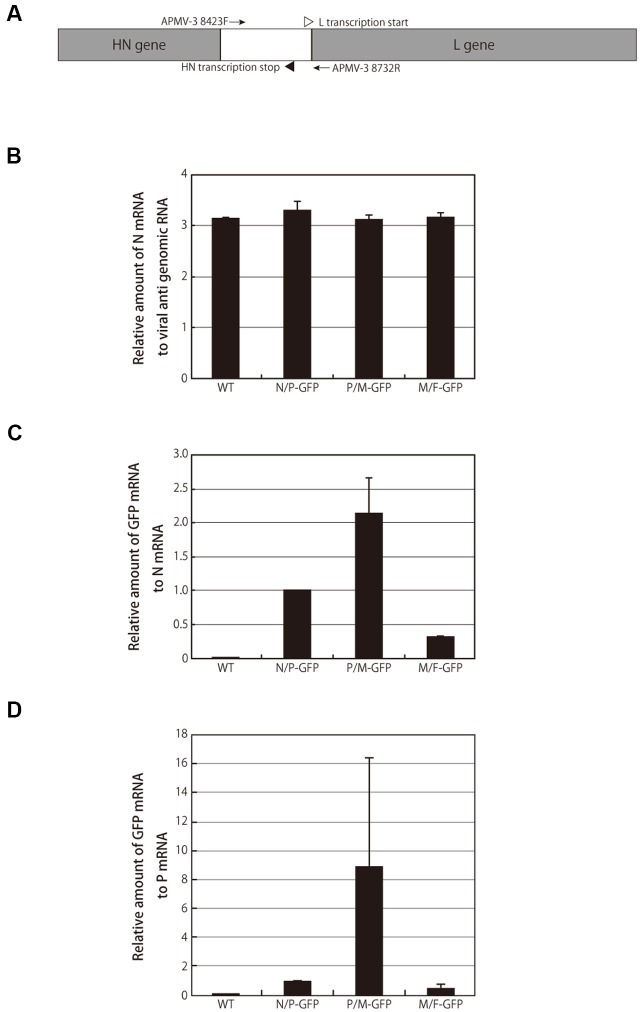
**Green fluorescent protein mRNA transcription. (A)** Schematic representation of a part of the APMV-3 genome with HN and L ORFs highlighted in gray. Primers used in the experiments are represented at the corresponding positions of the genome as arrows. Site of L transcription start and HN transcription stop are represented as open and closed triangles, respectively. **(B)** The relative amount of N mRNAs to anti-genomic RNAs detected in the cells infected with indicated viruses at 24 h p.i. by two-step qRT-PCR is shown as bar graph. **(C,D)** The relative amount of GFP mRNAs detected in the cells infected with indicated viruses at 24 h p.i. by two-step qRT-PCR is shown as bar graph. The amount of RNAs detected in N/P-GFP virus-infected cells was set to 1.

To determine if the highest expression level of GFP in the cells infected with the P/M-GFP recombinant virus was due to a higher replication level, we compared the mRNA transcript levels of viral N and GFP genes in the cells infected with recombinant APMV-3 viruses expressing GFP at 24 h p.i.. The mRNA of the infected cells was subjected to two-step qRT-PCR using the primer sets described in the “Materials and Methods” section (**Table 1** and **Figure 3A**), and the amount of N mRNA was standardized by the (+)-sense anti-genomic RNA. There was no significant difference in the ratio of N mRNA to anti-genomic RNA among these recombinant viruses (**Figure 3B**), indicating that the viral RNA replication and the mRNA transcription occurred at similar rates among these viruses (**Figure 2B**). We further evaluated the levels of GFP mRNA over those of N and P mRNAs. Consistent with the protein expression results shown in **Figure 2**, the N/GFP and P/GFP mRNA ratios in the M/F-GFP virus-infected sample were 3.3- and 2.2-fold lower than those in the N/P-GFP virus-infected samples, respectively. The GFP mRNA transcription levels detected in the P/M-GFP virus-infected cells were 2.0- and 8.8-fold greater than those of the N/P-GFP virus-infected cells, respectively (**Figures 3C,D**). These results indicate that the higher level of GFP expression observed in the P/M-GFP virus-infected cells compared to the N/P-GFP virus-infected cells is due to a higher transcription level of the GFP gene, suggesting that the P-M gene junction is the optimal insertion site for foreign gene expression in the APMV-3 vector.

## Discussion

A number of paramyxoviruses have been used as vaccine vectors (Kim and Samal, 2016; Liang et al., 2016; Seki and Matano, 2016; Swett-Tapia et al., 2016). Paramyxoviruses are good vaccine vectors because they do not have a DNA phase in their life cycle and therefore do not integrate into host cell DNA. In general, paramyxoviruses have a natural tropism for the respiratory tract and thus induce high levels of local and systemic immunity. In comparison to positive-strand RNA viruses, the genome of paramyxoviruses is genetically stable. Among paramyxoviruses, NDV possesses several additional characteristics that make it an attractive vaccine vector for human and animal pathogens. NDV is an avian virus; therefore, the entire human and non-avian animal populations are free of antibodies to NDV. NDV is highly safe in humans and in non-avian species due to natural host range restriction. NDV replicates well *in vivo* and elicits a potent immune response against the foreign antigens.

Although NDV is an ideal vaccine vector for humans and animals, it has several disadvantages for use as a vaccine vector in chickens. It may not be very effective as a vaccine vector in chickens less than 3 weeks old due to presence of maternal antibodies to NDV or in older chickens due to pre-existing neutralizing antibodies to NDV. Furthermore, the commonly used attenuated NDV strain LaSota is slightly pathogenic to young chickens and to chicken embryos. Therefore, it can not be used as a vaccine vector for 1-day old chicks and for *in ovo* vaccination. In order to circumvent these issues, there is a need for an alternative vaccine vector that is resistant to NDV antibodies and apathogenic to chicken embryos and to 1-day old chicks.

All strains of NDV belong to APMV serotype 1, but there are an additional 12 APMV serotypes (Afonso et al., 2016; Goraichuk et al., 2016). These other APMV serotypes are not well studied because they do not cause serious disease in chickens or turkeys. Among other APMV serotypes except for APMV-1, APMV-3 is attractive as a vaccine vector in chickens because it is highly safe in 1-day old chicks and in embryos and replicates well *in vitro* and *in vivo* (Kumar et al., 2010, 2011; Kim et al., 2012). We have previously reported that APMV-3 did not replicate at the same level as APMV-1, but replicated well enough to induce a protective immune response (Kumar et al., 2011). This study has found that APMV-3 vectors expressing NDV antigens are capable of inducing protective immunity against NDV, but it did not determine the optimal insertion site in the APMV-3 genome to express a foreign gene for vaccine purpose. There is concern that the efficacy of APMV-3 vector could be reduced by the pre-existing antibodies to NDV because there is antigenic cross reaction between APMV-3 and NDV (Nayak et al., 2012). We disagree because APMV serotypes were determined by serum neutralization test and therefore, NDV antibodies would not neutralize APMV-3. However, the replication of APMV-3 vector needs to be tested in chickens having NDV antibodies.

In order for APMV-3 to be effective as a vaccine vector, the foreign gene must be expressed at a high level. The level of foreign gene expression depends on several factors, such as size and sequence of the foreign genes, the properties of the foreign proteins, and the location of the foreign gene in the genome of the virus. It is important to know the optimal insertion site in the genome of APMV-3 for foreign gene expression. In NNS RNA viruses, gene expression is controlled primarily at the level of transcription based on the position of the gene relative to the single transcriptional promoter located at the 3′ end of the genome (Lamb and Parks, 2013). The best position for the highest level of expression of a foreign gene would be the 3′ most position in the APMV-3 genome. However, in the closely related NDV it was found that the promoter-proximal N and P junction was not the optimal insertion site but the P and M junction was the optimal insertion site for foreign gene expression (Zhao et al., 2015). Therefore, we aimed to find the optimal insertion site for foreign genes in the APMV-3 vector in order to achieve high level of expression.

In this study, we tested several aspects of foreign gene expression using APMV-3 vector by inserting an additional transcriptional unit at each of the gene junctions. In order to avoid any effect on expression level caused by the length and sequence of the foreign gene, the GFP gene was inserted at the 3′ non-coding regions of each gene. This allowed us to compare the expression levels of the foreign protein at each position and the effect of the foreign gene insertion at each position on growth characteristics of APMV-3.

Our results showed that the level of GFP expression did not strictly follow the 3′-to-5′ attenuation rule of NNS RNA virus. The highest level of GFP gene expression was observed when the gene was placed between the P and M genes. Although the level of GFP gene expression was high when it was placed between the N and P genes, it was lower than that when the GFP gene was placed between the P and M genes. Insertion of the GFP gene into the downstream M-F, F-HN, and HN-L gene junctions resulted in lower levels of GFP expression compared to those of the upstream positions. Interestingly, the level of GFP expression was higher when the GFP gene was placed between F and HN gene compared to when it was placed between the upstream M and F genes. Similar non-linear attenuation of foreign gene expression has also been observed in NDV (Zhao et al., 2015). However, in contrast with NDV, foreign gene mRNA abundance was also shown in a non-linear attenuation manner and they were correlated with their expression levels. In addition, transcription of viral gene of rabies virus strain HEP-Flury was reported not to follow the 3′-to-5′ attenuation rule, where as rabies virus strain ERA was reported to strictly follow the 3′-to-5′ attenuation rule (Morimoto et al., 2011).

Growth of the recombinant APMV-3 viruses was insignificantly affected by the insertion of the GFP gene except when it was inserted between the N and P genes. Insertion of a foreign gene between the N and P genes of NDV also affected NDV replication (Zhao et al., 2015). Although the exact reason for the observed delayed growth when a foreign gene is inserted between the N and P genes is not known, it may be due to alteration of the ratio of N and P proteins (Lamb and Parks, 2013).

## Conclusion

Our data suggest that the P and M gene junction of APMV-3 is the optimal insertion site for high level expression of a foreign gene. It is not known whether the P and M gene junction is the optimal insertion site for all foreign genes or just the GFP gene, thus requires further investigation. The findings reported here have important implication for the use of APMV-3 as a vaccine vector. APMV-3 may be a better vaccine vector than NDV for avian pathogens.

## Author Contributions

Conceived and designed the experiments: AY and SS. Performed the experiments: AY. Analyzed the data: AY and SS. Contributed reagents/materials/analysis tools: SS. Wrote the manuscript: AY and SS.

## Conflict of Interest Statement

The authors declare that the research was conducted in the absence of any commercial or financial relationships that could be construed as a potential conflict of interest.
